# Standardized Prevalence Ratios for Chronic Hepatitis C Virus Infection Among Adult Japanese Hemodialysis Patients

**DOI:** 10.2188/jea.JE20090043

**Published:** 2010-01-05

**Authors:** Masaki Ohsawa, Karen Kato, Kazuyoshi Itai, Kozo Tanno, Yosuke Fujishima, Ryuichiro Konda, Akira Okayama, Koichi Abe, Kazuyuki Suzuki, Motoyuki Nakamura, Toshiyuki Onoda, Kazuko Kawamura, Kiyomi Sakata, Takashi Akiba, Tomoaki Fujioka

**Affiliations:** 1 Department of Hygiene and Preventive Medicine, Iwate Medical University, Morioka, Japan; 2 Department of Urology, Iwate Medical University, Morioka, Japan; 3 The First Institute of Health Service, Japan Anti-Tuberculosis Association, Tokyo, Japan; 4 Division of Gastroenterology and Hepatology, Department of Internal Medicine, Iwate Medical University, Morioka, Japan; 5 Division of Cardiovascular Medicine, Nephrology and Endocrinology, Department of Internal Medicine, Iwate Medical University, Morioka, Japan; 6 Iwate Health Service Association, Morioka, Japan; 7 Division of Blood Purification, Kidney Center, Tokyo Women’s Medical University, Tokyo, Japan

**Keywords:** hepatitis C virus infection, hemodialysis, standardized prevalence ratio (SPR), population-based study, cross-sectional analysis

## Abstract

**Background:**

Many studies have estimated the prevalence of anti-hepatitis C virus (HCV) antibody among hemodialysis (HD) patients; however, the prevalence of HCV core antigen—which indicates the presence of chronic HCV infection—is not known.

**Methods:**

Standardized prevalence ratios (SPRs) for anti-HCV antibody and HCV core antigen among HD patients (*n* = 1214) were calculated on the basis of data from the general population (*n* = 22 472) living in the same area.

**Results:**

The prevalences of anti-HCV antibody and HCV core antigen were 12.5% and 7.8%, respectively, in male hemodialysis patients, and 8.5% and 4.1% in female hemodialysis patients. The SPRs (95% confidence interval) for anti-HCV antibody and HCV core antigen were 8.39 (6.72–10.1) and 12.9 (9.66–16.1), respectively, in males, and 5.42 (3.67–7.17) and 8.77 (4.72–12.8) in females.

**Conclusions:**

The prevalences of chronic HCV infection among male and female HD patients were 13-fold and 9-fold, respectively, those of the population-based controls. Further studies should therefore be conducted to determine the extent of chronic HCV infection among HD patients in other populations and to determine whether chronic HCV infection contributes to increased mortality in HD patients.

## INTRODUCTION

The prevalence of hepatitis C virus (HCV) infection in hemodialysis patients is very high.^1^^–^^15^ Because hemodialysis patients are vulnerable to HCV infection due to the risk of HCV exposure associated with the dialysis procedure and blood transfusion,^16^^–^^18^ infection control measures have been established to reduce the risks of HCV infection. Tests for detecting antibodies to HCV were first licensed by the Food and Drug Administration (FDA) in 1990^19^ and are now used worldwide. The risk of HCV infection due to dialysis and blood transfusion has therefore dramatically decreased.

The estimated prevalence of HCV infection in hemodialysis patients, although lower than in the past, remains high in developed countries in Europe, despite measures to prevent transmission of HCV.^13^^,^^20^^,^^21^ It has been suggested that HCV infection independently contributes to increased mortality among hemodialysis patients.^14^^,^^22^^–^^26^ In order to reduce mortality associated with HCV infection among hemodialysis patients, the prevalence of HCV infection and the factors that predispose hemodialysis patients to HCV infection require immediate investigation.

The prevalence of anti-HCV antibody among hemodialysis patients has been estimated in many studies, but the prevalence of chronic HCV infection is not known. In general, patients who are anti-HCV antibody-positive include those who are chronically infected and those who have recovered from infection. However, all patients who are HCV core antigen-positive are considered chronically infected. Therefore, it is necessary to test for both anti-HCV antibody and HCV core antigen to accurately assess the extent of chronic HCV infection in hemodialysis patients.

We investigated the prevalences of anti-HCV antibody and HCV core antigen in hemodialysis patients. We then compared these prevalences with those of the general population and examined associations between the prevalences and hemodialysis vintage.

## SUBJECTS AND METHODS

### Subjects

**Figure 1.**
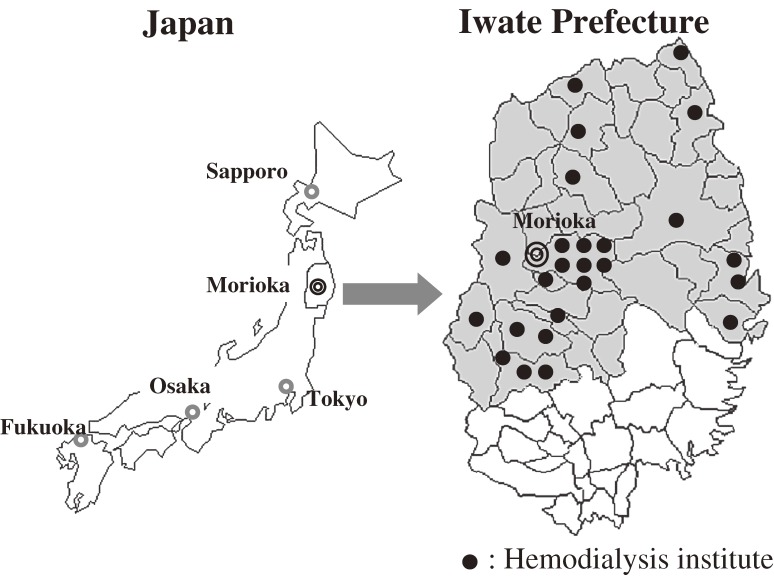
Maps of the KAREN Study area. The maps show the location of Morioka (the capital of Iwate Prefecture), in northeastern Honshu island. The KAREN Study area (shaded area) covers approximately two-thirds of Iwate Prefecture, and includes 26 hemodialysis facilities; only 1 facility (in which 7 patients were treated) was not included in the study. Closed circles indicate the sites of the hemodialysis facilities.

**Figure 2.**
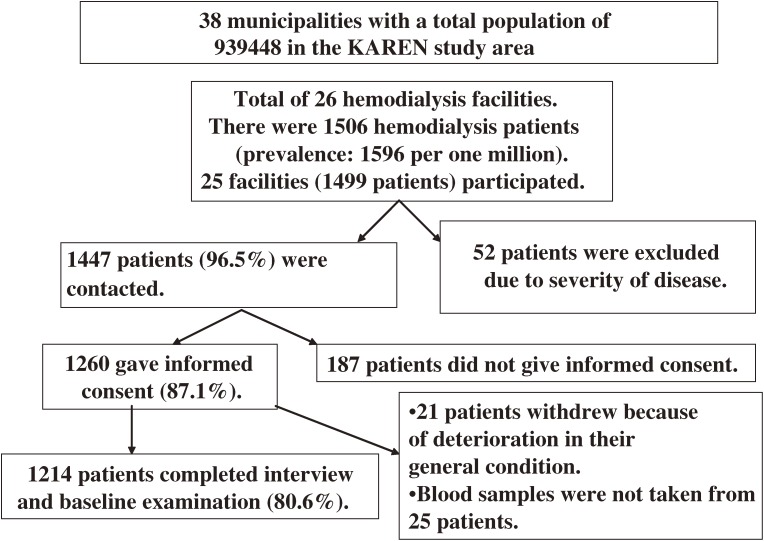
Flow chart for selecting subjects participating in the KAREN Study.  A total of 1506 adult patients were undergoing hemodialysis in 26 institutes in the study area. We were able to contact 1447 patients (96.5%); an additional 52 patients were excluded because of the severity of their condition. A total of 1260 patients (87.1%) gave written informed consent for participation in the study. Of these, 1214 (80.6%) completed the baseline examination.

We have conducted the “Kaleidoscopic Approaches to patients with end-stage RENal disease Study” (the KAREN Study) since 2003 in northern Japan (Figure 1). The KAREN Study is a population-based prospective study designed to determine the effects of risk factors on cardiovascular morbidity and mortality in end-stage renal disease (ESRD) patients.^27^ A total of 1214 adult hemodialysis patients (80.6% of the total number of hemodialysis patients in the study area; age 22 to 95 years; 779 males and 435 females) are included in the KAREN Study. Figure 2 shows a flow chart of the procedure for selecting subjects participating in the KAREN Study.

Control subjects were recruited from the general population living in the same area, and comprised 22 474 participants (7650 men and 14 824 women) who underwent annual health check-ups in Iwate Prefecture and HCV screening tests in 2005.

This study was approved by the Medical Ethics Committee of Iwate Medical University and was conducted in accordance with the guidelines of the Declaration of Helsinki.

### Measurements

The initial investigations in the KAREN Study were conducted from June 2003 through March 2004. These consisted of a questionnaire, review of medical records, measurements of blood pressure and anthropometric data, and blood tests. Anthropometrical examinations and blood pressure measurements were performed in a consistent manner. Self-administered questionnaires were used to collect individual information on demographic characteristics, history of cardiovascular disease, use of medication, alcohol consumption, and smoking status.^27^

Two medical doctors and 8 nurses visited 25 medical facilities and reviewed patients' medical records and treatment regimens. They recorded patient characteristics, such as age, sex, past history, family history, date when hemodialysis was initiated, length of hemodialysis sessions, number of hemodialysis sessions per week, prescribed dry weight, interdialysis weight gain at the beginning of the week, cause of ESRD, diabetes status, comorbid conditions, current medications, and history of other hemodialysis treatment.^27^

In the present study, information on anti-HCV antibody serology testing was collected by reviewing medical charts. All anti-HCV antibody serology tests at the 25 medical facilities were performed by using a second- or third-generation assay.

Predialysis blood sampling was performed by dialysis nursing staff immediately before beginning hemodialysis sessions. Blood samples were drawn from arteriovenous fistulae or grafts through hemodialysis cannulae into vacuum tubes. The blood samples were transported to a laboratory (Mitsubishi Kagaku Bio-Clinical Laboratories, Inc., Morioka branch office), and biochemical measurements and combined blood counts were performed on the same day. Residual sera of each sample were collected and stored at −80 °C in our laboratory.

**Figure 3.**
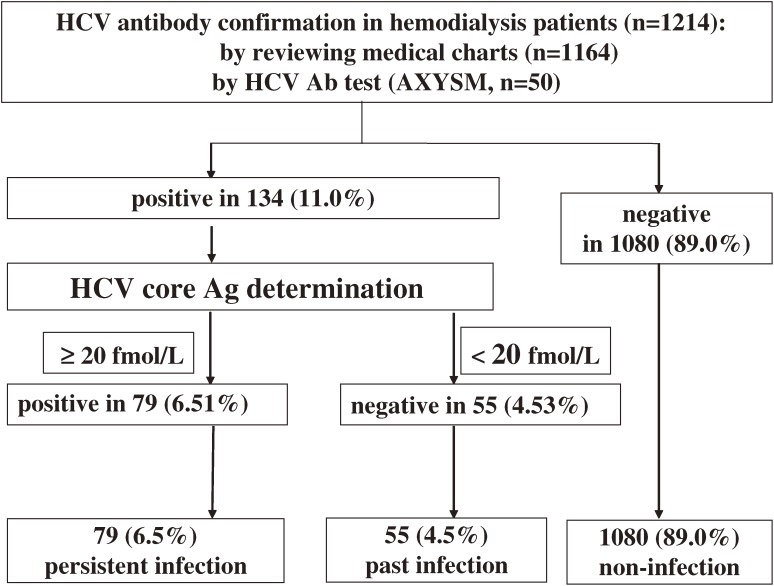
Flow chart of HCV antibody and HCV core antigen screening in hemodialysis patients in the KAREN Study.  Information on HCV serology tests was not collected from 50 subjects in the KAREN Study. For those 50 subjects, we defrosted frozen serum samples and performed HCV antibody tests using Architect HCV (Abbott, Japan). A total of 134 subjects (11.0%) were positive for HCV antibody. HCV core antigen tests were then performed for those subjects. A total of 79 were positive for HCV core antigen and were classified with persistent HCV infection (6.0%).

Results of anti-HCV antibody tests could not be obtained from 50 patients upon reviewing their medical charts. Frozen serum samples from those patients were unfrozen and anti-HCV antibody tests were performed using a second-generation assay (Architect HCV, Abbott, Japan). Frozen samples from patients who were positive for anti-HCV antibody (as confirmed by chart review or by HCV antibody determination using frozen samples) were unfrozen and HCV core antigen tests were performed using the Chemiluminescent Enzyme Immunoassay (CLEIA). Quantitative determination of HCV-RNA by reverse transcription polymerase chain reaction (RT-PCR) was not performed in hemodialysis patients who were positive for anti-HCV antibody and negative for HCV core antigen (Figure 3).

**Figure 4.**
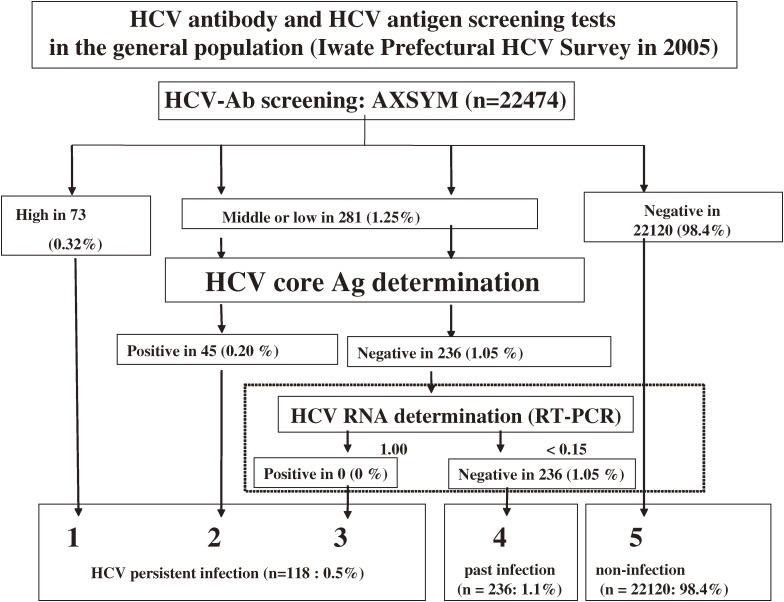
Flow chart of HCV antibody and HCV core antigen screening in population-based controls (Iwate Prefectural HCV survey in 2005).  There were 22 474 participants who underwent annual health check-ups and HCV screening. A total of 354 subjects were positive for HCV antibody (1.57%). HCV core antigen tests were performed in subjects with low- or middle-range positivity for HCV antibody. A total of 45 were positive for HCV core antigen. HCV-RNA determination using the RT-PCR method was performed in 236 subjects, but none were positive. Ultimately, 118 subjects were classified with persistent HCV infection (0.53%).

The HCV screening survey of the general population was conducted in Iwate Prefecture in 2005. All samples were transported to a laboratory (Iwate Health Service Association), and HCV antibody serology tests were performed by using an enzyme immunoassay (AxSYM HCV Dynapack II, Abbott Japan). Additional HCV core antigen tests were also performed using CLEIA in subjects who were positive for HCV antibody. A total of 236 samples from participants who were positive for anti-HCV antibody and negative for HCV core antigen were then used for qualitative determination of HCV-RNA by RT-PCR (AMPLICOR TM HCV test, Roche, Figure 4).

### Statistical analysis

Hemodialysis patients and population-based control subjects were divided into sex- and age-specific groups (20–39, 40–49, 50–59, 60–69, and ≥70 years). Sex- and age-specific prevalences of anti-HCV antibody and HCV core antigen were determined both in hemodialysis patients and controls.

Among hemodialysis patients, the expected number of patients positive for anti-HCV antibody (or HCV core antigen) in each sex- and age-specific group was calculated by using the prevalence of each sex- and age-specific group from the population-based controls. The total number of expected patients positive for anti-HCV antibody (or HCV core antigen) among hemodialysis patients was calculated by summing the numbers of positive individuals expected in all age-specific groups. The ratio of the observed number of hemodialysis patients with anti-HCV antibody (or HCV core antigen) to the expected number was defined as the standardized prevalence ratio (SPR). We assumed that the data would have a Poisson distribution; therefore, the confidence intervals for the SPRs were estimated using standard errors.^28^

Hemodialysis patients were also divided into 6 groups according to dialysis “vintage” (length of time on dialysis): <6 months, 6 to 23 months, 2 to 4 years, 5 to 9 years, 10 to 14 years, or 15 years or longer. Prevalences of anti-HCV antibody and HCV core antigen in each group were estimated. Differences in prevalences by sex or dialysis vintage (vintage ≥10 years vs <10 years) were tested using the chi-square test. To examine whether each risk factor was independently associated with chronic HCV infection or past HCV infection, logistic regression analysis was performed using presence of chronic HCV infection or history of HCV infection as the dependent variable and age, sex, and dialysis vintage as explanatory variables. A *P* value less than 0.05 was considered statistically significant. All statistical analyses were performed using the SPSS software package (SPSS, Japan Inc., Version 14.0).

## RESULTS

Table 1 shows sex- and age-specific prevalences of anti-HCV antibody in hemodialysis patients and population-based controls. Among population-based controls, the prevalence of anti-HCV antibody increased with advancing age; however, no such association was observed among hemodialysis patients. A sex difference in the prevalence of anti-HCV antibody was not found in the population-based controls; however, among the hemodialysis patients, the prevalence of anti-HCV antibody was higher in men than in women (12.5% vs 8.5%, *P* < 0.05).

**Table 1.** Sex- and age-specific prevalences of anti-HCV antibody in hemodialysis patients and a general population

Age group	General population		HD patients	
	
	Total No.	HCV Ab-positive (%)	Total No.	HCV Ab-positive (%)
Men				
20–39	36	0 (0.0%)	52	4 (7.7%)
40–49	890	16 (1.8%)	96	13 (13.5%)
50–59	1564	14 (0.9%)	191	38 (19.9%)
60–69	3001	43 (1.4%)	233	27 (11.6%)
≥70	2159	50 (2.3%)	207	15 (7.2%)
total	7650	123 (1.6%)	779	97 (12.5%)

Women				
20–39	62	0 (0.0%)	22	0 (0.0%)
40–49	2662	22 (0.8%)	55	5 (9.1%)
50–59	3980	40 (1.0%)	121	5 (4.1%)
60–69	4927	87 (1.8%)	116	1 (0.9%)
≥70	3193	82 (2.6%)	121	11 (9.1%)
total	14 824	231 (1.6%)	435	37 (8.5%)

Abbreviations: HCV, hepatitis C virus; HD, hemodialysis; No., number; Ab, antibody.

The prevalence of anti-HCV antibody was considerably higher in hemodialysis patients than in controls. The SPR (95% CI) for anti-HCV antibody was 8.39 (6.72–10.1) in male hemodialysis patients and 5.42 (3.67–7.17) in female hemodialysis patients.

Table 2 shows sex- and age-specific prevalences of HCV core antigen in hemodialysis patients and population-based controls. A positive association between the prevalence of HCV core antigen and age was found in controls but not in hemodialysis patients. The prevalence of HCV core antigen was also higher in male hemodialysis patients than in female hemodialysis patients (7.8% vs 4.1%, *P* < 0.05). The SPR (95% CI) for HCV core antigen was 12.9 (9.66–16.1) in male hemodialysis patients and 8.77 (4.72–12.8) in female hemodialysis patients.

**Table 2.** Sex- and age-specific prevalences of HCV core antigen in hemodialysis patients and normal controls

Age group	General population		HD patients	
	
	Total No.	HCV core Ag-positive (%)	Total No.	HCV core Ag-positive (%)
Men				
20–39	36	0 (0.0%)	52	3 (5.8%)
40–49	890	8 (0.9%)	96	8 (8.3%)
50–59	1564	5 (0.3%)	191	32 (16.8%)
60–69	3001	16 (0.5%)	233	12 (5.2%)
≥70	2159	21 (1.0%)	207	6 (2.9%)
total	7650	50 (0.7%)	779	61 (7.8%)

Women				
20–39	62	0 (0.0%)	22	0 (0.0%)
40–49	2662	5 (0.2%)	55	2 (3.6%)
50–59	3980	5 (0.1%)	121	5 (4.1%)
60–69	4927	28 (0.6%)	116	4 (3.4%)
≥70	3193	30 (0.9%)	121	7 (5.8%)
total	14 824	68 (0.5%)	435	18 (4.1%)

Abbreviations: HCV, hepatitis C virus; HD, hemodialysis; No., number; Ag, antigen.

Table 3 shows prevalences of anti-HCV antibody and HCV core antigen by dialysis vintage. Male and female patients with longer hemodialysis vintages (10–14 years or ≥15 years) had high prevalences of anti-HCV antibody than did male and female patients with a dialysis vintage less than 10 years (*P* < 0.05). Male and female patients with a dialysis vintage of 15 years or more had extremely high prevalences of anti-HCV antibody. However, among the dialysis vintage subgroups, male patients with a dialysis vintage of 15 years or more had the highest prevalence of HCV core antigen.

**Table 3.** Prevalences of anti-HCV antibody and HCV core antigen among hemodialysis patients, stratified by hemodialysis vintage

HD vintage	No.	HCV Ab-positive (%)	HCV core Ag-positive (%)
Men			
<6 months	44	4 (9.1%)	3 (6.8%)
6–23 months	158	14 (8.9%)	8 (5.1%)
2–4 yrs	218	18 (8.3%)	10 (4.6%)
5–9 yrs	176	15 (8.5%)	7 (4.0%)
10–14 yrs	75	10 (13.3%)	8 (10.7%)
≥15 yrs	108	36 (33.3%)	25 (23.1%)
total	779	97 (12.5%)	61 (4.6%)

Women			
<6 months	18	1 (5.6%)	1 (5.6%)
6–23 months	74	4 (5.4%)	3 (4.1%)
2–4 yrs	129	8 (6.2%)	4 (3.1%)
5–9 yrs	109	8 (7.3%)	4 (3.7%)
10–14 yrs	49	3 (6.1%)	3 (6.1%)
≥15 yrs	56	13 (23.2%)	3 (5.4%)
total	435	37 (8.5%)	18 (4.1%)

Abbreviations: HCV, hepatitis C virus; HD, hemodialysis; No., number; Ab, antibody; Ag, antigen.

Both male and female patients in the 4 groups with the shortest dialysis vintage (ie, <10 years) had similar prevalences of HCV antibody, regardless of dialysis vintage (approximately 9% in male hemodialysis patients and 5% in female hemodialysis patients in each of the 4 groups).

Table 4 shows the odds ratios attributable to each factor for having chronic HCV infection or past HCV infection. Male sex and dialysis vintage were independently associated with a higher prevalence of chronic HCV infection. The prevalence of chronic HCV infection among male hemodialysis patients was double that of female patients. However, only hemodialysis vintage was independently associated with an increased prevalence of past HCV infection.

**Table 4.** Odds ratios for each risk factor for past or chronic HCV infection

Risk factor	Chronic HCV infection			Past HCV infection		
	
	OR	95%CI	*P*	OR	95%CI	*P*
Age (per 1 year increase)	0.99	(0.97–1.01)	0.484	1.02	(0.99–1.05)	0.107
Male sex	1.99	(1.14–3.44)	0.014	1.06	(0.60–1.89)	0.843
Dialysis vintage (per 1 year increase)	1.09	(1.06–1.12)	<0.001	1.09	(1.06–1.13)	0.006

Odds ratios and their 95% confidence intervals were estimated by logistic regression analysis.

Abbreviations: OR, odds ratio; CI, confidence interval.

## DISCUSSION

In this study, we analyzed the prevalences of HCV antibody and HCV core antigen in adult hemodialysis patients. We estimated SPRs for both anti-HCV antibody and HCV core antigen among hemodialysis patients, and compared these estimates to those of the general population living in the same area.

Patients who are positive for HCV core antigen all have chronic HCV infection, whereas patients with anti-HCV antibody include those who have recovered from HCV infection, as well as those with chronic HCV infection. In a general population, patients who have recovered from HCV infection never develop liver cirrhosis or hepatocellular carcinoma (HCC) due to HCV, whereas patients with chronic HCV infection will develop liver cirrhosis or HCC 20 to 30 years after initial infection.^29^Therefore, in a general population, information regarding chronic HCV infection is more important than information on anti-HCV antibody.

In their study of Tunisian hemodialysis patients, Bouzgarrou et al reported that an HCV core antigen assay based on the HCV-RNA test had high sensitivity and high specificity; however, they were unable to provide an accurate estimate of the prevalence of chronic HCV infection and past HCV infection because of the large number of missing cases.^30^

Table 5 shows prevalences of anti-HCV antibody and chronic HCV infection (positivity for HCV core antigen or HCV RNA) in several studies with large sample sizes.^6^^,^^8^^–^^15^^,^^24^ Hmaied reported the prevalences of both anti-HCV antibody and HCV-RNA.^11^ The proportion of patients with HCV-RNA among patients with anti-HCV antibody was 70% in their study, and this proportion is similar to that of patients with HCV core antigen among patients with anti-HCV antibody in our study; it is also similar to the proportion of patients with chronic infection among all patients with HCV infection in the general population.^31^

**Table 5.** Prevalences of anti-HCV antibody and HCV core antigen (or RNA) among hemodialysis patients from various countries

Country	Author or name of study	Sample size	HCV Ab-positive (%)	Positive for HCV Ag or RNA (%)	Years tested
Japan	Washio^15^	540	24.3	—	1990
	Nakayama^24^	1470	18.8	—	1993
	DOPPS^8^	not obtained	19.9	—	1997–2001
	Kumagai^6^	1882	—	12.9^a^	1999–2003
	*KAREN*	1214	11.0	6.5^b^	2003–2004
United States	DOPPS^8^	not obtained	14.4	—	1997–2001
	Da Vita^14^	13 664	11.6	—	2001–2004
Belgium	Jadoul^13^	629	6.8	—	2000
France	DOPPS^8^	not obtained	14.7	—	1997–2001
Germany	Hinrichsen^9^	2796	7.0	—	1996–1997
United Kingdom	DOPPS^8^	not obtained	2.7	—	1997–2001
Italy	DOPPS^8^	not obtained	22.2	—	1997–2001
Iran	Shamshirsaz^10^	593	—	8.6^a^	2004^c^
Tunisia	Hmaied^11^	395	20	14^a^	2001–2003
Thailand	Luengrojanakul^12^	221	—	19.9^a^	1994

Abbreviations are the same as those used in Tables 1, 2, and 3. Italics indicate the present study.

Superscript numbers correspond to the reference used in the present study.

^a^, determined by HCV-RNA test by the PCR method; ^b^, determined by HCV core antigen test.

^c^, Not clearly described when blood sampling was performed (published in 2004).

We determined the prevalences of anti-HCV antibody and HCV core antigen in hemodialysis patients who were divided into 6 groups according to hemodialysis vintage. Patients with a hemodialysis vintage of 10 years or more had significantly higher prevalences of anti-HCV antibody and HCV core antigen than did patients with shorter hemodialysis vintages. Furthermore, patients with a hemodialysis vintage of 15 years or more had significantly higher prevalences of anti-HCV antibody than did other groups.

Since 1981, the Japanese Red Cross Blood Transfusion Service has excluded blood samples from donors with high serum ALT levels (≥36 KU/mL) in order to prevent transfusion of blood with non-A non-B hepatitis virus. Erythropoietin has been used clinically for treatment of anemia since 1986. In 1989, the Japanese Red Cross Blood Transfusion Service began using a first generation assay to screen blood donors for anti-HCV antibody.^32^ The timing of the introduction of these programs explains the relatively low prevalence of HCV infection among patients with a dialysis vintage less than 10 years and the extremely high prevalence of HCV infection among patients with a dialysis vintage of 15 years or more.

Choo and Kuo first developed a specific assay for HCV in 1989,^33^^,^^34^ and a second-generation ELISA, which was more sensitive than the first-generation ELISA, was developed in 1992 and became widely used as a clinical diagnostic tool and for epidemiological and other investigative purposes. As a result, the risk of nosocomial HCV infection has dramatically decreased among hemodialysis patients who started hemodialysis treatment after 1992. Our results showing a high prevalence of HCV infection among patients with a hemodialysis vintage of 10 years or more are consistent with the fact that risks for HCV infection have been reduced by the development and widespread use of HCV assays.

However, as compared to the general population, patients with a hemodialysis vintage of less than 10 years had a significantly higher prevalence of HCV infection, even though they would be expected to be at low risk of HCV infection due to blood transfusion and dialysis. This cross-sectional analysis also showed that prevalences were similar among the groups of patients with a dialysis vintage less than 10 years (ie, <6 months, 6–23 months, 2–4 years, 5–9 years), which suggests that most hemodialysis patients with HCV infection became infected before initiation of hemodialysis treatment, and that only a few patients with HCV infection developed the infection after initiation of hemodialysis treatment.

The incidence rate of HCV infection among hemodialysis patients is reported to be lower than 0.5 percent per year,^6^^,^^35^ indicating that the very high prevalence of HCV infection among hemodialysis patients is not entirely due to the elevated risk of nosocomial infection associated with dialysis therapy. There are several possible pathways for HCV transmission before initiation of hemodialysis. Patients with renal failure may have a high prevalence of HCV infection, regardless of the severity of renal failure, or, alternatively, patients with HCV infection may have a high prevalence of renal failure. It has been shown that HCV is associated with an increased prevalence of renal insufficiency.^36^ Renal diseases associated with HCV infection may also contribute to the high prevalence of HCV infection among patients with kidney disease.^37^

Another possible explanation is that patients with mild-to-moderate renal failure (ie, patients with chronic kidney disease) tend to develop ESRD after HCV infection, which may contribute to the high prevalence of HCV among patients with ESRD. Two studies have shown that HCV infection contributed to an increased risk of developing ESRD.^38^^,^^39^ If HCV infection does indeed contribute greatly to the development of ESRD, better prevention and treatment strategies for HCV infection should not only decrease liver disease-related mortality, they should also decrease the development of ESRD and its related mortality in patients with CKD and in the general population.

Although there was no sex-based difference in the prevalence of HCV infection in the general population, the prevalences of anti-HCV antibody and HCV core antigen were higher in male hemodialysis patients than in female hemodialysis patients. This suggests that male hemodialysis patients are at greater risk for HCV infection, perhaps due to the presence of predisposing factors for HCV infection.

Male hemodialysis patients with a long hemodialysis vintage (≥10 years) had a high rate of chronic HCV infection (70%: the percentage of patients who were positive for HCV core antigen among those were positive for anti-HCV antibody); however, female patients with a similarly long hemodialysis vintage had a lower rate of chronic HCV infection (37.5%). Male sex was independently associated with a high prevalence of HCV core antigen in logistic regression analysis. These data suggest that male hemodialysis patients have a greater risk of HCV infection, and a greater risk of persistent HCV infection, than do female hemodialysis patients.

Thomas et al reported that the spontaneous clearance rate of HCV among female patients was 1.58 times that of male subjects; however, the finding was of only marginal statistical significance.^40^ Women are less likely to be regular alcohol drinkers.^27^^,^^31^ In addition, they have higher levels of serum HDL cholesterol^27^^,^^41^ and perhaps other unknown protective factors. This may attenuate their risks of initial and chronic HCV infection, and may explain the observed sex-based differences.

Another possible explanation is that women who had recovered from HCV were selectively registered in the study because of a very high mortality rate for women with chronic HCV infection. However, to our knowledge, no studies have shown that female patients with chronic HCV infection have a higher mortality rate than that of patients who have recovered from HCV infection.

One major feature of this study is the long dialysis vintage of the participants. Mean dialysis vintage of the study participants exceeded 7 years; mean dialysis vintage was only approximately 3 years in reports from the United States and Europe.^42^ The generous medical insurance reimbursement system for Japanese dialysis patients and the high quality of hemodialysis treatment, which includes legal controls that strictly restrict re-use of a dialyzer, may have contributed to the longevity of hemodialysis patients. More than 20% of patients in the present study had long dialysis vintage (≥10 years), and long dialysis vintage was associated with a high prevalence of HCV infection in our study.

Since hemodialysis patients have a short life expectancy, there are few cases in which liver cirrhosis or HCC develops long after initiation of hemodialysis. Nakayama and Fabrizi found that hemodialysis patients who were anti-HCV antibody-positive had higher rates of liver disease-related deaths.^24^^,^^26^ However, the authors did not reveal whether an elevated mortality rate among hemodialysis patients with anti-HCV antibody was totally attributable to the increase in liver disease-related deaths. It is necessary to determine which cause of death contributes to the increase in mortality among hemodialysis patients with HCV infection.

This study was based on data from a population-based study and the sample size was sufficient to satisfy our objectives. Indeed, the large sample size of population-based controls living in the same area is one of the strengths of the study. However, several limitations to our study should be noted. The cross-sectional design of the present study cannot prove causal relationships. In addition, the lack of HCV-RNA data on the hemodialysis subjects who were positive for HCV antibody and negative for HCV core antigen is a major limitation in our study. It is possible that hemodialysis patients who are negative for HCV core antigen nevertheless have very low levels of HCV-RNA; however, the possibility of missing such cases in the present study is very low because, among the population-based controls, none were simultaneously positive for both HCV-RNA and HCV antibody and negative for HCV core antigen (Figure 4). Therefore, we believe that the results of the study were not distorted by lack of data regarding HCV-RNA. A history of blood transfusion is a strong predisposing factor for HCV infection. Thus, lack of information about past history of blood transfusion is also a major limitation. In addition, people who did not participate in the annual health check-ups may have been in poor health and might have had liver disease. This would have resulted in an underestimation of HCV infection in the general population and overestimation of the SPR for HCV among hemodialysis patients.

In conclusion, the prevalences of chronic HCV infection in male and female hemodialysis patients are 13 times and 9 times those of men and women in the general population. Further studies should therefore be carried out to determine the extent of chronic HCV infection in hemodialysis patients in other populations and to determine whether chronic HCV infection contributes to increased mortality in hemodialysis patients.

## References

[1] Petrosillo N , Gilli P , Serraino D , Dentico P , Mele A , Ragni P , Prevalence of infected patients and understaffing have a role in hepatitis C virus transmission in dialysis . Am J Kidney Dis. 2001;37:1004–10 10.1016/S0272-6386(05)80017-4 11325683

[2] Zampieron A , Jayasekera H , Elseviers M , Lindley E , DeVos JY , Visser R , European study on epidemiology and management of hepatitis C virus (HCV) infection in the haemodialysis population. Part 3: prevalence and incidence . EDTNA ERCA J. 2006;32:42–4 16700168 10.1111/j.1755-6686.2006.tb00445.x

[3] Montella M , Crispo A , Grimaldi M , Angeletti C , Amore A , Ronga D , Prevalence of hepatitis C virus infection in different population groups in southern Italy . Infection. 2005;33:9–12 10.1007/s15010-005-4036-1 15750753

[4] Tokars JI , Finelli L , Alter MJ , Arduino MJ National surveillance of dialysis-associated diseases in the United States, 2001 . Semin Dial. 2004;17:310–9 10.1111/j.0894-0959.2004.17339.x 15250925

[5] Espinosa M , Martn-Malo A , Ojeda R , Santamara R , Soriano S , Aguera M , Marked reduction in the prevalence of hepatitis C virus infection in hemodialysis patients: causes and consequences . Am J Kidney Dis. 2004;43:685–9 10.1053/j.ajkd.2003.12.030 15042545

[6] Kumagai J , Komiya Y , Tanaka J , Katayama K , Tatsukawa Y , Yorioka N , Hepatitis C virus infection in 2,744 hemodialysis patients followed regularly at nine centers in Hiroshima during November 1999 through February 2003 . J Med Virol. 2005;76:498–502 10.1002/jmv.20389 15977246

[7] Bergman S , Accortt N , Turner A , Glaze J Hepatitis C infection is acquired pre-ESRD . Am J Kidney Dis. 2005;45:684–9 10.1053/j.ajkd.2004.12.014 15806471

[8] Fissell RB , Bragg-Gresham JL , Woods JD , Jadoul M , Gillespie B , Hedderwick SA , Patterns of hepatitis C prevalence and seroconversion in hemodialysis units from three continents: the DOPPS . Kidney Int. 2004;65:2335–42 10.1111/j.1523-1755.2004.00649.x 15149347

[9] Hinrichsen H , Leimenstoll G , Stegen G , Schrader H , Fölsch UR , Schmidt WE ; PHV Study Group Prevalence and risk factors of hepatitis C virus infection in haemodialysis patients: a multicentre study in 2796 patients . Gut. 2002;51:429–33 10.1136/gut.51.3.429 12171969 PMC1773370

[10] Shamshirsaz AA , Kamgar M , Bekheirnia MR , Ayazi F , Hashemi SR , Bouzari N , The role of hemodialysis machines dedication in reducing Hepatitis C transmission in the dialysis setting in Iran: a multicenter prospective interventional study . BMC Nephrol. 2004;5:13 10.1186/1471-2369-5-13 15469615 PMC529260

[11] Hmaied F , Ben Mamou M , Saune-Sandres K , Rostaing L , Slim A , Arrouji Z , Hepatitis C virus infection among dialysis patients in Tunisia: incidence and molecular evidence for nosocomial transmission . J Med Virol. 2006;78:185–91 10.1002/jmv.20526 16372289

[12] Luengrojanakul P , Vareesangthip K , Chainuvati T , Murata K , Tsuda F , Tokita H , Hepatitis C virus infection in patients with chronic liver disease or chronic renal failure and blood donors in Thailand . J Med Virol. 1994;44:287–92 10.1002/jmv.1890440313 7531758

[13] Jadoul M , Poignet JL , Geddes C , Locatelli F , Medin C , Krajewska M , The changing epidemiology of hepatitis C virus (HCV) infection in haemodialysis: European multicentre study . Nephrol Dial Transplant. 2004;19:904–9 10.1093/ndt/gfh012 15031348

[14] Kalantar-Zadeh K , Kilpatrick RD , McAllister CJ , Miller LG , Daar ES , Gjertson DW , Hepatitis C virus and death risk in hemodialysis patients . J Am Soc Nephrol. 2007;18:1584–93 10.1681/ASN.2006070736 17429053

[15] Washio M , Ikeda M , Okuda S , Makita Y , Hirakata H , Kanai H , Hepatitis C virus antibody among chronic hemodialysis patients and predialysis renal failure patients . J Epidemiol. 1993;3:7–10

[16] Dussol B , Berthezéne P , Brunet P , Roubicek C , Berland Y Hepatitis C virus infection among chronic dialysis patients in the south of France: a collaborative study . Am J Kidney Dis. 1995;25:399–404 10.1016/0272-6386(95)90100-0 7532916

[17] Furusyo N , Kubo N , Nakashima H , Kashiwagi K , Etoh Y , Hayashi J Confirmation of nosocomial hepatitis C virus infection in a hemodialysis unit . Infect Control Hosp Epidemiol. 2004;25:584–90 10.1086/502443 15301031

[18] Sypsa V , Psichogiou M , Katsoulidou A , Skoutelis G , Moutafis S , Hadjiconstantinou V , Incidence and patterns of hepatitis C virus seroconversion in a cohort of hemodialysis patients . Am J Kidney Dis. 2005;45:334–43 10.1053/j.ajkd.2004.09.021 15685512

[19] Alter M, Evatt B, Margolis H, Biswas R, Epstein J, Feinstone S, et al. CDC. Public Health Service inter-agency guidelines for screening donors of blood, plasma, organs, tissues, and semen for evidence of hepatitis B and hepatitis C. Morbidity and Mortality Weekly Report. Atlanta, GA: Centers for Disease Control and Prevention, 1991: 1–17.

[20] Schneeberger PM , Keur I , van Loon AM , Mortier D , de Coul KO , van Haperen AV , The prevalence and incidence of hepatitis C virus infections among dialysis patients in the Netherlands: a nationwide prospective study . J Infect Dis. 2000;182:1291–9 10.1086/315869 11023452

[21] Gallego E , López A , Pérez J , Llamas F , Lorenzo I , López E , Effect of isolation measures on the incidence and prevalence of hepatitis C virus infection in hemodialysis . Nephron Clin Pract. 2006;104:c1–6 10.1159/000093252 16685138

[22] Stehman-Breen CO , Emerson S , Gretch D , Johnson RJ Risk of death among chronic dialysis patients infected with hepatitis C virus . Am J Kidney Dis. 1998;32:629–34 10.1016/S0272-6386(98)70027-7 9774125

[23] Pereira BJ , Natov SN , Bouthot BA , Murthy BV , Ruthazer R , Schmid CH , Effects of hepatitis C infection and renal transplantation on survival in end-stage renal disease. The New England Organ Bank Hepatitis C Study Group . Kidney Int. 1998;53:1374–81 10.1046/j.1523-1755.1998.00883.x 9573555

[24] Nakayama E , Akiba T , Marumo F , Sato C Prognosis of Anti-Hepatitis C Virus Antibody-Positive Patients on Regular Hemodialysis Therapy . J Am Soc Nephrol. 2000;11:1896–902 11004221 10.1681/ASN.V11101896

[25] Kalantar-Zadeh K , McAllister CJ , Miller LG Clinical characteristics and mortality in hepatitis C-positive haemodialysis patients: a population based study . Nephrol Dial Transplant. 2005;20:1662–9 10.1093/ndt/gfh895 15905194

[26] Fabrizi F , Takkouche B , Lunghi G , Dixit V , Messa P , Martin P The impact of hepatitis C virus infection on survival in dialysis patients: meta-analysis of observational studies . J Viral Hepat. 2007;14:697–703 17875004 10.1111/j.1365-2893.2007.00868.x

[27] Ohsawa M , Kato K , Itai K , Onoda T , Konda R , Fujioka T , Cardiovascular Risk Factors in Hemodialysis Patients: Results from Baseline Data of Kaleidoscopic Approaches to Patients with End-stage Renal Disease Study . J Epidemiol. 2005;15:96–105 10.2188/jea.15.96 15930806 PMC7851064

[28] Altman D. Practical statistics for medical research. London: Chapman & Hall, 1991.

[29] National Institutes of Health National Institutes of Health Consensus Development Conference Statement: Management of hepatitis C: 2002--June 10–12, 2002 . Hepatology. 2002;36:S3–20 10.1002/hep.1840360703 12407572

[30] Bouzgarrou N , Fodha I , Othman SB , Achour A , Grattard F , Trabelsi A , Evaluation of a total core antigen assay for the diagnosis of hepatitis C virus infection in hemodialysis patients . J Med Virol. 2005;77:502–8 10.1002/jmv.20485 16254976

[31] Afdhal NH The natural history of hepatitis C . Semin Liver Dis. 2004;24 supple 2:3–8 10.1055/s-2004-832922 15346240

[32] Washio M Blood-borne viral infection in hemodialyis units: special reference to hepatitis B virus, hepatitis C virus and human T-lymphotrophic virus type 1 . Nippon Koshu Eisei Zasshi. 1998;45:960–7 (in Japanese) 9893464

[33] Choo QL , Kuo G , Weiner AJ , Overby LR , Bradley DW , Houghton M Isolation of a cDNA clone derived from a blood-borne non-A, non-B viral hepatitis genome . Science. 1989;244(4902):359–62 10.1126/science.2523562 2523562

[34] Kuo G , Choo QL , Alter HJ , Gitnick GL , Redeker AG , Purcell RH , An assay for circulating antibodies to a major etiologic virus of human non-A, non-B hepatitis . Science. 1989;244(4902):362–4 10.1126/science.2496467 2496467

[35] Izopet J , Sandres-Sauné K , Kamar N , Salama G , Dubois M , Pasquier C , Incidence of HCV infection in French hemodialysis units: a prospective study . J Med Virol. 2005;77:70–6 10.1002/jmv.20415 16032714

[36] Dalrymple LS , Koepsell T , Sampson J , Louie T , Dominitz JA , Young B , Hepatitis C virus infection and the prevalence of renal insufficiency . Clin J Am Soc Nephrol. 2007;2:715–21 10.2215/CJN.00470107 17699487

[37] El-Serag HB , Hampel H , Yeh C , Rabeneck L Extrahepatic manifestations of hepatitis C among United States male veterans . Hepatology. 2002;36:1439–45 12447870 10.1053/jhep.2002.37191

[38] Crook ED , Penumalee S , Gavini B , Filippova K Hepatitis C is a predictor of poorer renal survival in diabetic patients . Diabetes Care. 2005;28:2187–91 10.2337/diacare.28.9.2187 16123488

[39] Tsui JI , Vittinghoff E , Shlipak MG , Bertenthal D , Inadomi J , Rodriguez RA , Association of hepatitis C seropositivity with increased risk for developing end-stage renal disease . Arch Intern Med. 2007;167:1271–6 10.1001/archinte.167.12.1271 17592100

[40] Thomas DL , Astemborski J , Rai RM , Anania FA , Schaeffer M , Galai N , The natural history of hepatitis C virus infection: host, viral, and environmental factors . JAMA. 2000;284:450–6 10.1001/jama.284.4.450 10904508

[41] Dreux M , Pietschmann T , Granier C , Voisset C , Ricard-Blum S , Mangeot PE , High density lipoprotein inhibits hepatitis C virus-neutralizing antibodies by stimulating cell entry via activation of the scavenger receptor BI . J Biol Chem. 2006;281:18285–95 10.1074/jbc.M602706200 16675450

[42] Leavey SF , McCullough K , Hecking E , Goodkin D , Port FK , Young EW Body mass index and mortality in ‘healthier’ as compared with ‘sicker’ haemodialysis patients: results from the Dialysis Outcomes and Practice Patterns Study (DOPPS) . Nephrol Dial Transplant. 2001;16:2386–94 10.1093/ndt/16.12.2386 11733631

